# Repeated Acetylcholine Receptor Antibody-Concentrations and Association to Clinical Myasthenia Gravis Development

**DOI:** 10.1371/journal.pone.0114060

**Published:** 2014-12-02

**Authors:** Anne Taraldsen Heldal, Geir Egil Eide, Fredrik Romi, Jone Furlund Owe, Nils Erik Gilhus

**Affiliations:** 1 Department of Clinical Medicine, University of Bergen, Bergen, Norway; 2 Centre of Clinical Research, Haukeland University Hospital, Bergen, Norway; 3 Department of Global Public Health and Primary Care, University of Bergen, Bergen, Norway; 4 Department of Neurology, Haukeland University Hospital, Bergen, Norway; Istanbul University, Turkey

## Abstract

**Introduction:**

We aimed to examine the longitudinal association between Myasthenia Gravis (MG) clinical severity and concentration of acetylcholine receptor (AChR)-antibodies to evaluate if AChR-antibody variations correlate to disease severity. A positive AChR-antibody test is specific for MG.

**Material and Methods:**

All patients from western Norway who had two or more AChR- antibody tests in the period 1983–2013 were identified. The Myasthenia Gravis Foundation of America (MGFA) Clinical Classification was used to grade disease development. Multiple ordinal logistic regression analysis was used to estimate a possible predictive effect for AChR-antibody concentration on MGFA classification result.

**Results:**

In 67 patients two or more AChR-antibody tests with a corresponding MGFA-score were performed, with a total of 309 tests. 56 patients were treated with immunosuppressive drugs and 11 by pyridostigmine only. There was a positive association between concentration of AChR-antibodies and longitudinal MGFA-score for the subgroup with immunosuppressive treatment, but not for those treated with pyridostigmine only. This association between AChR-antibody concentration and MGFA score declined with increasing time since onset (p = 0.005 for the interaction of group×time×concentration).

**Conclusions:**

For MG patients with immunosuppressive treatment, repeated AChR-antibody measurements give information about clinical development, and can therefore be of support in therapeutic decisions.

## Introduction

Myasthenia gravis (MG) is an autoimmune neuromuscular disease, with an incidence of 7–16 per million per year [1], [2]. The disorder is caused by antibodies binding to components in the neuromuscular junction [3], impairing neuromuscular transmission. In 85 percent of cases, the antibodies bind to the postsynaptic nicotinic acetylcholine receptor (AChR), termed anti-AChR MG [4]. The antibodies reduce the number of functional AChR by cross-binding the receptors with increased degradation [5], [6], lysis of postsynaptic membrane by complement activation [7], and by direct blockade [8]. In some patients, AChR- antibodies are detectable in a sensitive cell-based assay only [9]. 5–8 percent of the anti-AChR negative MG patients, have MG induced by antibodies to muscle-specific tyrosine kinase (MuSK) [10], [11], and in 3–9 percent to low-density lipoprotein receptor-related protein 4 (LRP4) [12]–[14].

The loss of functional AChRs causes fluctuating skeletal muscle weakness, fatigability, and improvement by rest. Ptosis and diplopia are frequent onset symptoms [15], [16]. Respiratory muscles can also be affected and lead to myasthenic crisis [17]. The fluctuation during the day and the variable predominance of affected muscle groups makes it difficult to score these patients for symptom severity. The Myasthenia Gravis Foundation of America (MGFA) has developed a uniformly accepted grading system [18] (Table 1), mainly developed for clinical trials, but also widely used in the clinical management of MG patients.

**Table 1:** Myasthenia Gravis Foundation of America Clinical Classification [18].

Class	Clinical symptoms
I	Any ocular weakness
II	Mild Weakness. May also have ocular muscle weakness of any severity
II A	Predominantly affecting limb, axial muscles, or both. May also have lesser involvement of oropharyngeal, respiratory muscles or both
II B	Predominantly affecting ororpharyngeal, respiratory muscles, or both. May also have lesser or equal involvement of limb, axial muscles or both
III	Moderate weakness affecting other than ocular muscles. May also have ocular muscle weakness of any severity
III A	Predominantly affecting limb, axial muscles, or both. May also have lesser involvement of oropharyngeal, respiratory muscles or both
III B	Predominantly affecting ororpharyngeal, respiratory muscles, or both. May also have lesser or equal involvement of limb, axial muscles or both
IV	Severe weakness affecting other than ocular muscles. May also have ocular muscle weakness of any severity
IV A	Predominantly affecting limb, axial muscles, or both. May also have lesser involvement of oropharyngeal, respiratory muscles or both
IV B	Predominantly affecting ororpharyngeal, respiratory muscles, or both. May also have lesser or equal involvement of limb, axial muscles or both
V	Defined by intubation, with or without mechanical ventilation, except when employed during routine postoperative management

Presence of AChR-antibodies is nearly 100 percent specific for MG [19]. The concentration of AChR-antibodies does not correlate with clinical status between individuals [20], [21]. Patients with mild disease may have high concentrations of AChR- antibodies, and vice versa. The association between intra-individual AChR- antibody concentration and clinical status is not clear. Studies evaluating this association are inconsistent, data are scarce, and most of the studies were conducted in the 1980s. There is a need for a prognostic marker to support therapeutic decisions regarding the intensity of the immunosuppressive therapy. An objective marker, such as AChR-antibody concentration, if associated with clinical state, should allow a more precise and consistent MG treatment. The number of repeated AChR-antibody tests taken of patients with a confirmed MG diagnosis increases markedly in some countries [1], [22]. This indicates that AChR-antibody concentration is widely used to evaluate clinical status and prognosis even though the scientific evidence for this practice is lacking.

A recent study found a weak correlation between change in AChR-antibody concentration and clinical status [23]. They concluded that concentration of AChR-antibodies might be useful as a marker for non-response or inadequate immunotherapy. The study concluded nevertheless not to recommend AChR-antibodies as a general follow-up biomarker, mainly because the concentration of AChR-antibodies fell also in most of the patients who did not improve.

In our study, we examined the association between concentration of AChR-antibodies and MG clinical state in individual patients from a national cohort over time to assess whether repeated antibody measurements have any predictive value for therapeutic decisions.

## Materials and Methods

### Ethics statement

Written consents were only obtained from patients alive as the Regional ethics committee allowed us to use patient data from deceased patients' next of kin without any further consent. The study was approved by the regional ethics committee (REK Vest, reference 2012/1480).

### Study population

Approximately 15 000 AChR- ntibody samples were analysed 1983–2013 at Haukeland University Hospital, Bergen, Norway. Patient information for all the tests included name, date of birth, name of referring hospital or doctor, and date of sample acquisition. All patients registered as living in the three counties Rogaland, Hordaland, and Sogn og Fjordane (western Norway) with two or more AChR-antibody tests were included in this study. Western Norway had 269 anti-AChR MG patients examined in the included time period. A total of 185 of these had more than one AChR-antibody test performed. Eighty-five patients were deceased and still included; 98 patients were alive on 1 November 2012 (study start), and two patients were impossible to trace. The patients alive were contacted in writing, informed and given consent form to sign. Signed consent was obtained from 82 patients (positive response rate 84 percent). Twentyfour of the deceased and 43 of those still alive had two or more AChR- antibody tests combined with a MGFA Clinical Classification. These 67 patients constituted our total sample. Fifty-six of the included patients had immunosuppressive treatment (immunosuppressive MG group), whereas 11 were treated with pyridostigmine only (pyridostigmine MG group).

Twenty-three patients had early-onset MG (debut <50 years), 26 had late-onset MG (debut >50 years). Fourteen had a thymoma, and five patients had pure ocular MG. Four patients (6.0%) had experienced a myasthenic crisis. Thirty-one patients were thymectomised.

### AChR-antibody assay

All AChR-antibody assays for patients from Western Norway were performed in a single laboratory at Haukeland University Hospital during the whole period. In the period 1983–1994, human striated muscle obtained at the Department of Surgery was used as antigen and AChR- antibody concentrations were given in arbitrary units (U/L). From 1994, a commercial kit has been used using AChR from human muscles as antigen in a radioimmunoassay (IBL-Hamburg GmbH, Germany). Concentrations are given in nmol/L. All samples with a concentration >0.4 nmol/L were defined as AChR-antibody positive [24]. All patient serum samples were stored at 4°C and analysed within 3 weeks. Four times a year, the laboratory participates in a control trial assessment to ensure the analytic quality. In the present study, only tests analysed after 1994 were included. Time for MG-onset was in this study defined as date of first positive AChR-antibody sample. The AChR-antibody sample was defined as valid if obtained one month before or after an MGFA-score [25].

### MGFA Clinical Classification

Myasthenia Gravis Foundation of America (MGFA) Clinical Classification [18] was used to evaluate the clinical status of the patients (Table 1). This classification was designed to identify MG subgroups with distinct clinical features or severity of the disease that could indicate different prognoses or responses to therapy. MGFA Clinical Classification was developed to accommodate the need for a universally accepted classification, and divides MG into five classes. In this study we did not subdivide into A or B, according to localisation of weakness. Patients with no symptoms were scored as 0. The MGFA-score was derived from structured notes in the neurological journal that included distinct clinical features of ocular-, limb- and/or bulbar weakness. We then identified all the AChR-antibody tests and linked the test with the corresponding MGFA-score. The MGFA Clinical Classification was therefore performed blinded to AChR-antibody concentration. Initially, and according to this assessment of the patients, two observers independently classified the first medical note of 33 randomly picked patients [26].

### Statistics

Descriptive statistics are reported as mean, median, standard deviation, minimum and maximum as appropriate. Inter-examiner agreement of the MGFA-score was quantified using the kappa coefficient and tested with McNemar-Bowker's test.

Clinical evaluations and AChR- antibody measurements were done at irregular time intervals for each patient. Therefore time since first test was analysed both as a continuous variable and divided into four quartiles for each patient. The first-quartile comprised 0.24 years (0–3 months), the second- quartile comprised 0.24–1.85 years (3–19 months), the third-quartile comprised 1.85 to 4.93 years (19–37 months), and the fourth-quartile comprised the rest of the time period (37 months-18 years). In the quartile model, the variation within each quartile was missed, but associations over time with regular intervals became more precisely elucidated. Multiple ordinal logistic regression using generalised estimating equations, correcting for repeated measures in the same individuals (intra-individual analyses) by assuming an exchangeable correlation structure, was used to estimate a possible predictive effect of AChR- antibody concentration (nmol/L) and time since onset on MGFA-score [27]. We especially tested for a change of effect of AChR-antibody concentration on MGFA-score over time since onset was different for the pyridostigmine and the immunosuppressed MG groups (test of time×AChR×MG group interaction). Results are reported as odds ratios (OR) and 95 percent confidence intervals (CI). SPSS 21 was used for all statistical analyses.

## Results

A total of 67 patients were included with 309 measurements of AChR- antibodies and with a corresponding MGFA -score. The immunosuppressed MG group constituted 56 of the 67 patients, with 272 antibody measurements and a corresponding MGFA-score. The pyridostigmine MG group constituted 11 patients, and with 37 AChR- antibody measurements with a corresponding MGFA-score. The number of measurements for each patient ranged from 2 to 13, with an average of 4.5 (Table 2). Mean MGFA-score was 2.0 for the immunosuppressed MG group and 1.9 for the pyridostigmine MG group (Table 3). The overall median MGFA-score was 2 in the first three quartiles and 1 in the fourth quartile, with the mean value decreasing from 2.0 to 1.3 over time (Table 4). Four patients had experienced a myasthenic crisis, three females and one male, all with late-onset MG. For all the 309 AChR-antibody tests from the 67 included MG patients, the concentrations ranged from 0 to >300 nmol/L, with a mean value between 19.1 and 27.2 nmol/L (Table 4). The mean concentration did not differ in the three first quartiles, but was decreased in the fourth (F-test p-value = 0.020 from mixed linear regression accounting for correlation between repeated measures within patients). The median AChR- antibody concentration was 14.4 for the immunosuppressed MG group, and 10.8 for pyridostigmine MG group. Inter-examiner agreement for MGFA was Kappa (K) 0.84 (almost perfect) (standard error (SE) 0.74, 95% confidence interval (CI): (0.70–0.99) [28]. Total percent of agreement was 88 percent. McNemar-Bowker's test showed no disagreement symmetry (p = 0.368). A high MGFA-score was more probable in the first quartile, and the score decreased significantly with increasing time since onset (Wald-p = 0.036 from unadjusted ordinal regression of MGFA on time quartiles). This illustrates how well-treated patients with MG improve over time. The immunosuppressed and the pyridostigmine MG groups differed significantly regarding the association between AChR-antibody concentration and MGFA-score over time since onset (three-way interaction in ordinal logistic regression Wald-p = 0.005). After further adjustment for gender, subclass and thymectomy this interaction was only marginally significant (p = 0.059). Results are given in Table 5 after some parameterization. Analyses with time since onset as a continuous variable gave p = 0.595 for the three-way interaction when we did not adjust for other variables, probably because the interaction is not linear by linear for the two continuous variables. When adjusting for the other variables the analyses never converged. We then analysed the two MG groups separately giving the following results.

**Table 2:** Distribution of number of AChR-antibody measurements with a corresponding MGFA-score in 67 patients 1983–2013.

Number of tests	Number of patients
2	17
3	13
4	13
5	6
6	5
7	4
8	2
9	3
10	1
11	0
12	1
13	2

**Table 3:** Demographic statistics at first antibody test of all MG patients (n = 67) with two or more positive AChR-antibody tests registered as living in western Norway 1983–2013 according to immunosuppressive (n = 56) and pyridostigmine (n = 11) therapy.

Variables	Immunosuppression		Pyridostigmine only		P-value
Age, *mean (SD)*	55.8	(17.9)	49.1	(24.1)	0.31^a^
**Sex, ** ***n (%)***					0.33^b^
Women	31	(55.4)	4	(36.4)	
Men	25	(44.6)	7	(63.6)	
**Subgroup, ** ***n (%)***					0.18^b^
Early-onset	16	(28.6)	6	(54.5)	
Late-onset	23	(41.1)	4	(36.4)	
Ocular	3	(5.4)	1	(9.1)	
Thymoma	14	(25.0)	0	(0.0)	
AChR-ab concentration, *median (IQR)*	14.4	(24.8)	10.8	(18.5)	0.55^a^
MGFA-score, *mean (SD)*	2.0	(1.4)	1.9	(1.2)	0.90^a^

*Abbreviations:* MG: Myasthenia gravis; SD: standard deviation; AChR: acetylcholine receptor; ab: antibody; MGFA: Myasthenia Gravis Foundation of America; IQR: interquartile range.

a)Wilcoxon Mann-Whitney test.

b)Exact chi-square test.

**Table 4:** Descriptive statistics for MGFA-score (1–5) and AChR-antibody concentration (nmol/L) in each quartile of time since onset for 309 tests of 67 MG patients at Haukeland University Hospital, Bergen, Norway 1983–2013.

		MGFA-score					AChR-antibody concentration				
Timea)	Number of tests	Median	Mean	SD	Min	Max	Median	Mean	SD	Min	Max
1^st^ quartile	77	2	2.0	1.3	0	5	12.0	26.6	46.1	0	>300
2^nd^ quartile	78	2	1.4	1.1	0	4	12.6	27.2	48.1	0	291
3^rd^ quartile	77	2	1.4	1.0	0	4	10.4	26.9	44.7	0	228
4^th^ quartile	77	1	1.3	1.0	0	5	6.5	19.1	33.3	0	208

*Abbreviations:* MGFA: Myasthenia Gravis Foundation of America; AChR: acetylcholine receptor; MG: Myasthenia gravis; SD: standard deviation.

a)1st quartile: 0–3 months; 2nd quartile: 3–19 months; 3rd quartile: 19–37 months; 4th quartile: 37 months–21 years.

**Table 5:** The effects of AChR-antibody concentrationa) on MGFA classification according to medication group and time since onset based on multiple ordinal logistic regression of 67 MG patients at Haukeland University Hospital, Bergen, Norway from 1983–2013.

Medication group	Odds ratios	95% CI
**Pyridostigmine MG group (11 patients)** b)		
1^st^ time quartile	0.03	(0.00, 1.25)
2^nd^ time quartile	1.05	(0.65, 1.70)
3^rd^ time quartile	0.69	(0.39, 1.22)
4^th^ time quartile	0.23	(0.07, 0.71)

Immunosuppressed MG group (56 patients)c)	Odds ratios	95% CI
1^st^ time quartile	4.10	(1.30,12.95)
2^nd^ time quartile	2.41	(1.11, 5.20)
3^rd^ time quartile	3.91	(1.71, 8.91)
4^th^ time quartile	2.48	(0.52,11.94)

*Abbreviations:* AChR: Acetylcholine receptor; MGFA: Myasthenia gravis Foundation of America (1–5); MG: Myasthenia gravis; CI: Confidence interval.

a)Odds ratio per 100 nmol/L.

b)Patients treated with pyridostigmine only.

c)Patients treated with immunosuppressive drugs.

### Immunosuppressed MG group

There was an association between changes in longitudinal AChR-antibody concentration and changes in MGFA-score in individual immunosuppressed MG patients. This was demonstrated by using both time since onset and AChR- antibody concentration as continuous variables. If the concentration of AChR-antibodies increased by 10 nmol/L, it was 10 percent more likely that the MGFA- score would worsen (common OR: 1.13, 95%: (1.06, 1.20). This association was also demonstrated by dividing time into four quartiles, showing no significant interaction between AChR- antibody concentration and MGFA-score on group level (Wald-p = 0.349). This indicates an effect of AChR-antibody concentration on the MGFA- score for the whole study period, though not significant for the last quartile (Table 5).

### Pyridostigmine MG group

In the group treated with pyridostigmine only, a positive association between AChR-antibody concentration and MGFA-score was not significant. Moreover, the effect of AChR-antibody concentration on MGFA-score also declined after the three first months (OR: from 1.05 in second quartile to 0.23 in the fourth quartile) when time since onset increased (Table 5). The declining effect of AChR-antibody concentration on MGFA- score over time was also demonstrated by using both time since onset and AChR- antibody concentration as continuous variables showing the significant interaction between the two (Wald-p = 0.033).

An interaction between AChR-antibody concentration and time since onset was neither found significant in the immunosuppressed MG group separately, nor in the pyridostigmine MG group. However, the overall analysis showed that they were statistically significant from each other. This means that there is a time-changing effect of AChR-antibody concentration on MGFA-score in both or one of the two groups, and we believe it to be most likely with such an effect in the immunosuppressed MG group.

## Discussion

There was an association between AChR-antibody concentration and MGFA-score when tested longitudinally in individual immunosuppressed MG patients and over many years. This indicates that repetitive determinations of AChR-antibody concentration can predict the clinical state for this group. As a valid biomarker, AChR-antibody tests probably reflect the degree of drug response and may help in decisions whether the clinician should modify or keep the immunosuppressive treatment unchanged regarding drug and drug dose. The clinical evaluation of MG disease status involves complex constructs of objective signs and subjective symptoms. For the past years, there has been put efforts into developing rating scales to evaluate the clinic in a more systematic way, but it is complicated to assess MG even with scales designed for this disorder [18], [29]. AChR-antibody testing should contribute in assessing the status of the patient. The association between MGFA-score and changes in AChR-antibody concentration was no longer significant after three years, probably due to a more stable disease after long-term immunosuppression. MG patients in this study improved over time, in line with previous studies [17], [30]. Only four patients (6%) underwent a myasthenic crisis, which is lower than expected [17], [31].

For the pyridostigmine MG group, there was no association between MGFA-score and concentration of AChR-antibodies for the first three quartiles. For the fourth quartile of the follow-up period, there was an association, which we, however, judge as a spurious effect. This indicates that repeated AChR-antibody tests are useful in monitoring MG patients on immunosuppressive treatment, but have less value in the follow-up of patients treated symptomatically with pyridostigmine only. The lack of association was demonstrated by a declining correlation between concentration of AChR-antibodies and MGFA-score with increasing time since onset. The difference between the immunosuppressed and the pyridostigmine MG-groups stresses the fact that the two groups should be handled differently regarding frequency of AChR-antibody testing and interpretation of the concentration. The reason why these two groups differ is probably due to the effect of immunosuppressive drugs on the autoantibody production. Corticosteroids, azathioprine and cyclosporine inhibit T-cell proliferation via several mechanisms, and thereby inhibit activation of B- cells and reduce the concentration of circulating AChR-antibodies. Cyclophosphamide and rituximab suppress B-cell activation and synthesis [32]–[35]. The AChR-antibodies destruct the muscle endplate, and a reduction of circulating AChR-antibodies improves the signaling between nerve and muscle [36], and thereby the MG symptoms.

The patients in this study constitute a population-based and representative cohort [1], with a very high rate of participation. However, only patients having two or more AChR-antibody concentration tests performed were included, and this may represent a selection bias. Patients with only one test may have had a stable disease in remission, and with less immunosuppressive treatment. All AChR-antibody samples were analysed at the same laboratory with the same commercial antibody assay method. This makes comparisons over time reliable.

We used the MGFA Clinical Classification [18] as a score to identify the course of the disease in the individual patient. There is an inherent imprecision when distinguishing between mild, moderate and severe muscle weakness. However, we found the MGFA- scoring suitable, confirmed by the validation. The near perfect validation result, outweighs to some extent the lack of prospective design. The classification is not sensitive to small changes due to symptom fluctuations during the day. For this study, it was important that the score was based on more robust and long-term clinical development.

This retrospective blinded and validated long- term follow- up study found that repeated AChR-antibody measurements are valuable to monitor response to immunosuppressive treatment in individual MG patients, and can be a support for therapeutic decisions.
